# Prevalence of isomeric plastomes and effectiveness of plastome super-barcodes in yews (*Taxus)* worldwide

**DOI:** 10.1038/s41598-019-39161-x

**Published:** 2019-02-26

**Authors:** Chao-Nan Fu, Chung-Shien Wu, Lin-Jiang Ye, Zhi-Qiong Mo, Jie Liu, Yu-Wen Chang, De-Zhu Li, Shu-Miaw Chaw, Lian-Ming Gao

**Affiliations:** 1 CAS Key Laboratory for Plant Diversity and Biogeography in East Asia, Kunming Institute of Botany, Chinese Academy of Sciences, Kunming, Yunnan 650201 China; 2 Germplasm Bank of Wild Species, Kunming Institute of Botany, Chinese Academy of Sciences, Kunming, Yunnan 650201 China; 3 Kunming College of Life Science, University of Chinese Academy of Sciences, Kunming, Yunnan 650201 China; 4 Biodiversity Research Center, Academia Sinica, Taipei, 11529 Taiwan

## Abstract

*Taxus* (yew) is both the most species-rich and taxonomically difficult genus in Taxaceae. To date, no study has elucidated the complexities of the plastid genome (plastome) or examined the possibility of whole plastomes as super-barcodes across yew species worldwide. In this study, we sequenced plastomes from two to three individuals for each of the 16 recognized yew species (including three potential cryptics) and *Pseudotaxus chienii*. Our comparative analyses uncovered several gene loss events that independently occurred in yews, resulting in a lower plastid gene number than other Taxaceous genera. In *Pseudotaxus* and *Taxus*, we found two isomeric arrangements that differ by the orientation of a 35 kb fragment flanked by “*trnQ*-IRs”. These two arrangements exist in different ratios within each sampled individual, and intraspecific shifts in major isomeric arrangements are first reported here in *Taxus*. Moreover, we demonstrate that entire plastomes can be used to successfully discriminate all *Taxus* species with 100% support, suggesting that they are useful as super-barcodes for species identification. We also propose that *accD* and *rrn16-rrn23* are promising special barcodes to discriminate yew species. Our newly developed *Taxus* plastomic sequences provide a resource for super-barcodes and conservation genetics of several endangered yews and serve as comprehensive data to improve models of plastome complexity in Taxaceae as a whole and authenticate *Taxus* species.

## Introduction

The plastid genomes (plastomes) of photosynthetic land plants are generally characterized by two unequal single-copy regions separated by a pair of canonical inverted repeats (IRs)^1^. However, coniferous plastomes lack the canonical IRs and show extensive rearrangements^2^. Recent comparative plastomics studies have revealed several lineage-specific and actively recombining IRs in conifers. For instance, Pinaceae-specific IRs are recombination substrates associated with the formation of distinct plastomic architectures^3,4^. In cupressophytes, lineage-specific IRs are able to mediate inversions at a subtle level, ultimately resulting in the existence of major and minor isomeric plastomes that differ based on how a particular region is oriented^5–9^. Shifts in major isomeric plastomes were observed at interspecific levels in a few Cupressaceous lineages^6,9^, but such shifts were absent in populations of *Calocedrus macrolepis*^9^. Nonetheless, the above observations were mainly based on Cupressaceae. Little is known about the shift in major isomeric plastomes at the intra- and inter-specific level in other cupressophyte families such as Taxaceae.

Plastomic sequences are excellent resources for resolving the tree of life^10^ and delimiting the species entity^11^. Plastids are predominantly inherited uniparentally^12^ and they behave as a single non-recombining locus, which provides a strong signal of phylogenetic history^13^. Plastid loci have been utilized widely as DNA barcodes for discriminating plant species^14^. A combination of two plastid loci (*matK* and *rbcL*) were suggested as the core barcode to discriminate species of land plants^15^; however, it generally could not distinguish closely related species or recently evolved species in most groups due to the lack of adequate variation among taxa^16^. Further specific barcodes could help improve this discriminatory power at the species level^17^. Therefore, the quest for improved barcodes with universal usage in plants is ongoing^18^. Although concerns were raised about the possibility of plastid introgression and hybridization^19–21^, many researchers advocated for the approach that uses whole plastomes as super-barcodes^22–24^. For example, the super-barcode approach was shown to successfully distinguish closely related species such as *Theobroma* spp. (Malvaceae)^25^, *Araucaria* spp. (Aruacariaceae)^26^, and *Echinacea* (Asteraceae)^27^, especially for taxonomically complex groups, e.g., *Camellia* spp. (Theaceae)^28^, *Epimedium* spp. (Berberidaceae)^29^, and *Fritillaria* spp. (Liliacae)^30^. The increased availability of plastome sequences and reduced cost of next generation sequencing (NGS) technology have recently sparked an interest in the versatility of plastomes. An approach combining the best use of single-locus barcodes and super-barcodes for efficient plant identification was suggested for selected groups of taxa, including specific barcodes that could distinguish closely related plants at the species and population levels^17^.

Taxaceae includes six genera (*Amentotaxus*, *Austrotaxus*, *Cephalotaxus*, *Pseudotaxus*, *Taxus*, and *Torreya*) and about 30 species of evergreen trees or shrubs, distributed mainly in the Northern Hemisphere^31,32^. This cupressophyte family likely diverged from its closest sister, Cupressaceae, during the Early Triassic^32,33^. *Taxus* (yews), the largest and most widespread genus in Taxaceae^34^, is famous for its high content of the anticancer compound taxol, a chemotherapeutic drug used in breast and lung cancer treatment^35^. However, yews have a complex and controversial taxonomic history due to their high degree of morphological similarity between species^36–39^. For example, Spjut^37^ recognized 24 species in *Taxus*, with 16 species and seven varieties in China, whereas Farjon^31^ only admitted 10 species in the genus, including only five in China. Recently, based on a global scale genetic and distribution analysis, Liu *et al*.^40^ approved a total of 15 *Taxus* species/lineages including the ten recognized by Farjon^31^, two by Spjut^37^, one by Möller *et al*.^39^ and two cryptics by Liu *et al*.^40,41^.

To date, reported plastomes are limited to few *Taxus* species, and super-barcodes have not been used to elucidate plastomes for *Taxus* species on a large scale. To this end, we sequenced the complete plastomes from all 16 recognized *Taxus* species (including three potential cryptics) and the sole species of *Pseudotaxus, P. chienii* (Cheng) Cheng, sampling three individuals per species except for the Huangshan type of *Taxus* and *P. chienii* because wild populations of them were unavailabile. Incorporating the previously elucidated plastomes of other Taxaceous genera, this study aims to address the following questions: 1) Do plastomic characteristics—in terms of genome size, gene content, nucleotide compositions, and structure—vary across the Taxaceae? 2) Are isomeric plastomes common in *Taxus*? If yes, do their relative abundances vary among species and/or populations? 3) Are whole plastome sequences suitable super-barcodes for discriminating yew species? If not, are there any special plastid genes/intergenic spacers that are promising barcoding loci for identifying yew species?

## Results

### The Plastome size and gene content vary across Taxaceae

**Figure 1:**
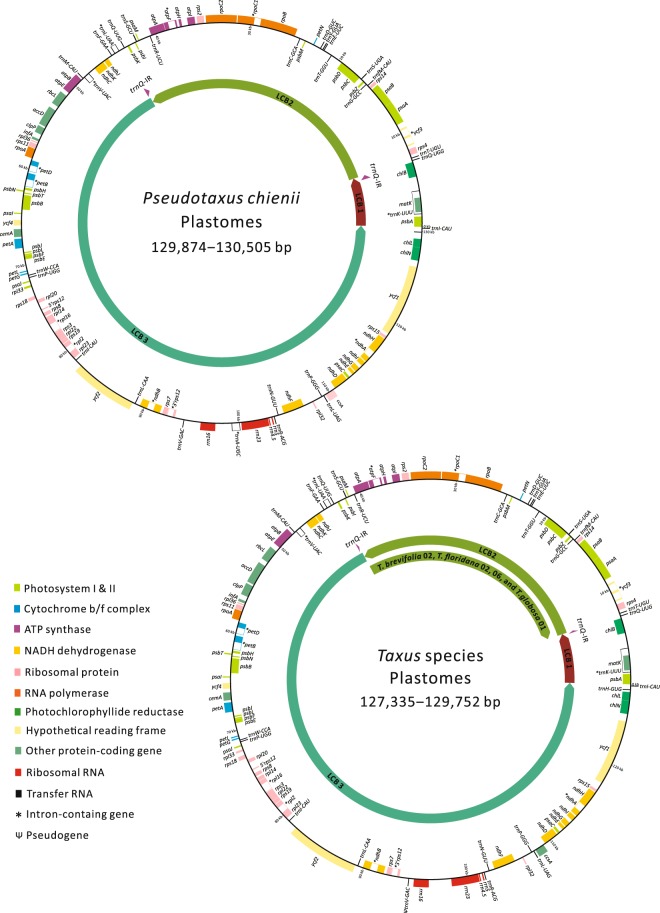
Circular maps of *Pseudotaxus* and *Taxus* plastomes. Colored boxes outside and inside the circle are genes in counterclockwise and clockwise transcribed directions, respectively. Locally co-linear blocks (LCBs 1, 2, and 3) between *Pseuodtaxus* and *Taxus* are indicated by the inner colored arrows with their relative orientations. Regions of *trnQ*-IRs are denoted by purple arrows along LCBs. Scale bars 10 kb in length are given with *psbA* as the starting point.

Plastomes were sequenced from 49 samples of all 16 recognized species of *Taxus* and *Pseudotaxus chienii*, with two to three individuals sampled per species. These 49 newly sequenced plastomes were assembled into circular molecules (Fig. 1), with an average sequencing coverage of 41 to 2,716 times (Table S1). They are deposited in GenBank under the accession numbers MH390441 to MH390489. The *Pseudotaxus* and *Taxus* plastomes are 129,874–130,505 bp and 127,335–129,752 bp long, respectively. They are shorter than previously reported plastomes in other Taxaceous genera (Table 1). The plastomic GC content across Taxaceae ranges from 34.6 to 35.9%, with *Taxus* being the lowest. A pair of short inverted repeats with a *trnQ-UUG* in each copy (hereafter called *trnQ*-IRs based on Guo *et al*.^6^) is also common in Taxaceae, with *Taxus* and *Amentotaxus* having the shortest and longest *trnQ*-IRs, respectively (Table 1).

**Table 1:** Comparison of plastomic characteristics across Taxaceae.

Genus	*Taxus*	*Pseudotaxus*	*Torreya*	*Amentotaxus*	*Cephalotaxus*
Plastome size (bp)	127,335‒129,752	129,874‒130,505	136,949‒137,075	136,430‒136,657	134,337‒136,196
GC content (%)	34.6‒34.8	35.2	35.4‒35.5	35.8‒35.9	35.1‒35.3
*trnQ*-IR length (bp)	216‒267	552	298	534‒564	544‒548
No. of genes^a^	114	116	121	120	116
**Variation in gene content** ^b^					
*rps16*	−	−	+	+	+
*trnA-UGC*	−	+	+	+	+
*trnV-GAC*	Ψ	+	+	+	+
*trnS-GGA*	−	−	+	+	+
*trnG-UCC*	−	−	+	+	+
*trnI-GAU*	−	−	+	+	+
*trnV-UAC*	+	+	+	+	−
*trnI-CAU*	+, +	+, +	+, +	+, +	+
*trnT-UGU*	+	+	+	+	Ψ
*trnN-GUU*	+	+	+, +	+, Ψ	+

^a^Shared genes: *accD, ccsA, cemA, clpP, infA, matK, rbcL, atpA, B, E, F, H, and I*; *chlB, L*, and *N*; *ndhA, B, C, D, E, F, G, H, I, J*, and *K*; *petA*, *B*, *D*, *G*, *L*, and *N*; *psaA*, *B*, *C*, *I*, *J*, *M*; *psbA*, *B*, *C*, *D*, *E*, *F*, *H*, *I*, *J*, *K*, *L*, *M*, *N*, *T*, and *Z*; *rpl2*, 14, 16, 20, 23, 32, 33, and 36; *rpoA*, *B*, *C1*, and *C2*; *rps2, 3, 4, 7, 8, 11, 12, 14, 15, 18*, and 19; *ycf1*, 2, 3, and 4; *rrn4*.5, *5*, 16, and 23; *trnC-GCA, trnD-GUC, trnE-UUC, trnF-GAA, trnfM-CAU, trnG-GCC, trnH-GUG, trnI-CAU, trnK-UUU, trnL-CAA, trnL-UAA, trnL-UAG, trnM-CAU, trnN-GUU, trnP-GGG, trnP-UGG, trnQ-UUG, trnQ-UUG, trnR-ACG, trnR-UCU, trnS-GCU, trnS-UGA, trnT-GGU, trnW-CCA*, and *trnY-GUA*.

^b^“−” absent; “+” present; “Ψ” pseudo; “+, +” duplicate; “+, Ψ” duplicate but one of them is pseudo.

The gene content varies from 114 to 121 genes per plastome, with the smallest and largest sets of genes being in *Taxus* and *Torreya*, respectively (Table 1). In total, 82 protein-coding genes, 4 ribosomal RNAs, and 25 transfer RNAs are shared across Taxaceae. Variation in gene content includes 1) pseudogenization of *trnV-GAC* in and loss of *trnA-UGC* from *Taxus*; 2) losses of *trnS-GGA*, *trnG-UCC*, *trnI-GAU*, and *rps16* from both *Pseudotaxus* and *Taxus*; 3) pseudogenization of *trnT-UGU* in *Cephalotaxus*, and losses of *trnV-UAC*, *trnV-GAC*, and a *trnI-CAU* copy from *Cephalotaxus*; and 4) duplication of *trnN-GUU* in *Torreya*, but one of the two *trnN-GUU* copies has become pseudogenized in *Amentotaxus*. Because *Amentotaxus* is phylogenetically close to *Torreya*^42^, duplication of *trnN-GUU* might predate the divergence of these two genera.

In addition to loss/duplication of genes, a specific extension of *clpP* was found in *Taxus*. *ClpP* encodes the caseinolytic protease, which contains 339‒537 and 224‒245 amino acids in *Taxus* and other Taxaceous genera, respectively. Therefore, there is great variation in the length of *clpP* at both intra- and inter-genus levels. As shown in the *clpP* alignment (Fig. 1S), a block of Glu (E)-rich insertions separates *Taxus* from other Taxaceous genera. Whether this Glu-rich insertion has implications in the fundamental function of *clpP* remains to be answered.

### Shifting major isomeric plastome arrangements at intraspecific levels

Three locally co-linear blocks (designated LCBs 1, 2, and 3) between *Pseudotaxus* and *Taxus* were identified in the same orientation with four exceptions (Fig. 1). The LCB 2 fragments of approximately 35 kb are inverted in four individuals—*T. brevifolia* 02, *T. globosa* 01, and *T. floridana* 02 and 05 (here named “arrangement B” following Guo *et al*.^6^)—and are not in the remaining 45 samples (designated “arrangement A”). Notably, these data also indicate intraspecific variation in the LCB 2 orientation in three taxa—*T. brecifolia*, *T. globosa*, and *T. floridana*. These LCB2 fragments are exclusively flanked by *trnQ*-IRs, regardless of the relative orientations (Fig. 1). Previously, *trnQ*-IRs were proposed to facilitate homologous recombination that generates isomeric plastome arrangements in *Cephalotaxus*^5^ and Cupressoideae^6,9^. Accordingly, if *trnQ*-IRs are active recombinant agents in *Pseudotaxus* and *Taxus*, we would expect arrangements A and B to coexist in each sample.

**Figure 2:**
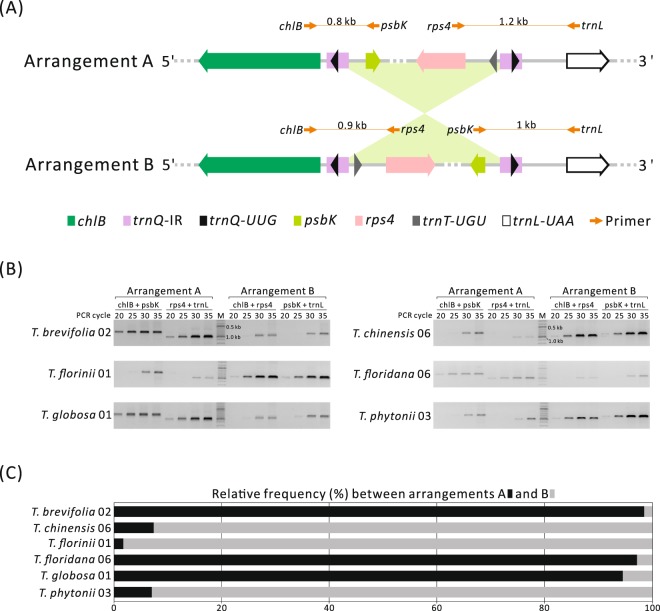
Verification of isomeric plastome arrangements. (**A**) Primer pairs designed to amplify specific regions of isomeric arrangements. (**B**) PCR results generated from 20, 25, 30, and 35 cycles of reactions to determine the major and minor isomeric arrangements in six exemplified *Taxus* accessions. The gel images were cropped from the full-length gels shown in Fig. S3. (**C**) Counts of Illumina pair-end reads that support isomeric arrangements. Stacked horizontal bars indicate the relative frequency between arrangements A and B.

Figure 2A shows that specific regions typifying isomeric arrangements A and B were detected by four primer pairs. For *T. brevifolia* 02, *T. floridana* 06, and *T. globosa* 01, specific amplicons of arrangement A were observed when 20 to 35 PCR cycles were used, while arrangement B appeared only when ≥30 PCR cycles were used (Fig. 2B). Conversely, the amplicons of arrangement B appeared earlier than those of arrangement A in *T. chinensis* 06, *T. florinii* 01, and *T. phytonii* 03. Similar PCR assays were performed for the remaining samples (Fig. S2). Collectively, our PCR results suggest that major and minor isomeric arrangements exist in both *Pseudotaxus* and *Taxus*.

The Illumina paired-end reads provide the other line of evidence that supports the coexistence of arrangements A and B in both *Pseudotaxus* and *Taxus*. For examples, 1,266 reads that spanned *trnQ*-IRs were gathered from *T. brevifolia* 02, of which 1,245 (98.3%) and 21 (1.7%) supported arrangements A and B, respectively (Fig. 2C); this is in agreement with the PCR result that suggests arrangement A being overwhelmingly abundant (Fig. 2B). Overall, coexistence of arrangements A and B was detected in 47 of the 49 samples, and the relative frequency of major arrangements was estimated to be 86.2% to 98.4% (Figs 2C; S2). None of the detected reads supported arrangement A in *P. chienii* 03 and *T. brevifolia* 03, possibly because 1) for the former, most reads (insertion size approximately 500 bp) were too short to include its entire *trnQ*-IR (552 bp), and 2) for the latter, there were not enough reads to detect the minor arrangement A, as its sequencing depth is the lowest among the examined samples (Table S1).

Taken together, data from PCRs and pair-end reads are consistent in supporting our plastome assemblies as well as the major isomeric arrangement B. As a consequence, intraspecific variation in plastomic organizations observed in *T. brevifolia*, *T. floridana*, and *T. globosa* (Fig. 1) suggests that shifts in major isomeric arrangements have occurred among populations.

### Whole plastomes as super-barcodes for discriminating yew species

**Figure 3:**
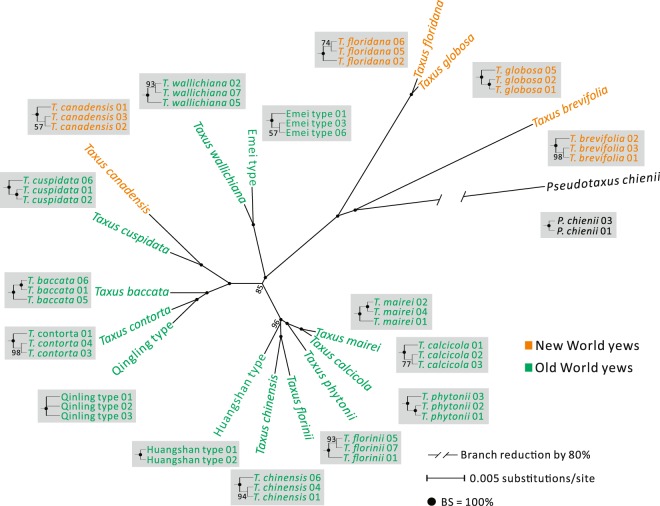
An ML tree constructed from whole plastome sequences with *Pseudotaxus* as the outgroup. Values (%) along branches were estimated from 1,000 bootstrap replicates. Inferred relationships of conspecific accessions are shown in grey boxes. The tree was condensed under a 50% majority rule.

As mentioned above, *Pseudotaxus* and *Taxus* share three LCBs. Alignments and concatenation of these three LCBs yielded a matrix containing 146,099 characters. An ML tree (Fig. 3) was inferred based on this plastomic matrix using *Pseudotaxus* as an outgroup. Of note, the four New World yews *T. brevifolia*, *T. globosa*, *T. floridana*, and *T. canadensis* did not form a monophyletic clade. Instead, *T. brevifolia* was placed as the earliest diverged yew, and *T. canadensis* was inferred to be more closely related to Old World yews than to other New World ones (Fig. 3). All Old World yews except *T. canadensis* were grouped into a clade separate from New World yews. Nonetheless, the conspecific accessions of all species, including those from the three potential cryptic types, were grouped into respective monophyletic clades each with 100% support, thereby suggesting that whole plastomes are effective super-barcodes for identifying yew species. The newly discovered species (Huangshan type) was well supported, which was close to *T. chinensis* and *T. florinii* (Fig. 3).

### Single genic loci as promising special barcodes for discriminating yew species

**Figure 4:**
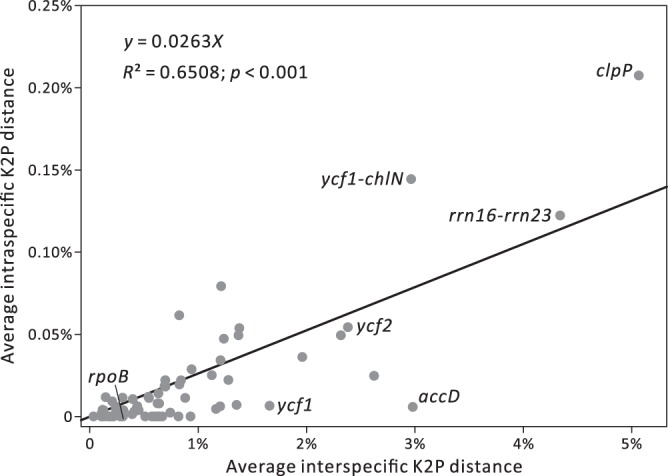
Positive correlation between intra- and inter-specific K2P distances among the 73 examined single genic and intergenic loci.

After excluding loci smaller than 400 bp, 73 syntenic loci, including 45 protein-coding genes and 28 intergenic spacers, were determined to assess their discriminatory power for yew species (Table S2). They are highly variable in length, with two extremes: the longest loci (*ycf1* ~6.7 kb long and *ycf2* ~7.3 kb) exceed the shortest locus *rps11* (~0.4 kb) by over 16-fold. For each locus, pairwise intra- and inter-specific K2P distances were estimated from 46 and 1,035 comparisons, respectively. Among the 73 single loci, the average intra- and inter-specific distances are positively correlated (Fig. 4), suggesting that intraspecific polymorphisms contributed to interspecific divergence increases in *Taxus* plastomes. In terms of both intra- and inter-specific distances, *clpP* (~1.2 kb) is a standout due to its Glu (E)-rich insertion. Two intergenic loci, *rrn16-rrn23* (~2 kb) and *ycf1-chlN* (~0.9 kb), also exhibit a great degree of intraspecific variation, implying that they may be useful in population genetics studies. We noted that *accD* (~2.2 kb) shows a conspicuous discrepancy between intra- and inter-specific K2P distances, with the former being smaller than the latter by approximately 298 times (Fig. 4). A discrete distribution between intraspecific variation and interspecific divergence (termed barcoding gap) is crucial for species discrimination (Hebert *et al*. 2004)^43^, so maximum intra- and minimum inter-specific distances were compared across all examined loci. Nonetheless, only three loci *accD*, *ycf1*, and *rpoB* (~3.3 kb) show no overlap (Table S2); this scarcity (3/76 = 3.95%) is attributed to the minimum interspecific distance of many loci being as low as 0%.

We applied the NJ tree method to evaluate the discriminatory rate for yew species using 73 single loci. Formation of a monophyletic clade of conspecific samples was treated as successful discrimination when the corresponding BS values were larger than 50%. A fully discriminatory rate was yielded in the trees inferred from *accD*, *ycf1*, *ycf2*, and *rrn16-rrn23*, but only two of them were diagnosed with a barcoding gap (Table S2). Although a barcoding gap existed in *rpoB*, the tree inferred from this gene did not discriminate species 100% successfully (Table S2). In contrast, all conspecific accessions formed monophyletic clades with 100% support in the tree inferred from a non-barcoding gap gene* ycf2* (Table S2).

## Discussion

New sequencing technologies are cost-effective and give data of previously unimaginable mass and quality. They have facilitated the sequencing of plastomes from numerous species^26,44–47^. In this study, 49 complete plastomes were obtained from *P. chienii* of the monotypic genus *Pseudotaxus* and 16 species of *Taxus*, including samples of three individuals of almost all species. Using *P. chienii* as the outgroup, our dataset, in terms of taxon sampling, is by far the most comprehensive among comparative plastomics across the genus *Taxus*. Based on these samples, we estimated the plastomic architecture variation at both intra- and inter-specific levels, examined the power of entire plastomes for discriminating species, and evaluated useful single loci as special DNA barcodes.

It is well accepted that the canonical IRs in plastomes are able to trigger intramolecular recombination to generate equal amounts of isomeric plastomes, one of which differs from the other by the relative orientation of its small single copy region^48,49^. Despite lacking the canonical IR, cupressophytes have evolved a diverse set of lineage-specific IRs capable of mediating inversions to form isomeric plastomes. For instance, isomeric plastomes associated with *trnQ*-IRs have been discovered in *Cephalotaxus*^5^ and Cupressaceae^6,9^. In *Sciadopitys* (Sciadopityaceae), the presence of *trnQ*-containing tandem repeats has led to the speculation that *trnQ*-IRs resulted from multistep rearrangements after a tandem duplication^50^. In addition, *Sciadopitys* contains specific IRs (called *rpoC2*-IRs) that are proven to be re-combinable^7^. The *trnN*-IRs that were proposed to be responsible for formation of isomeric plastomes^8^ are common in Podocarpaceae^51^. All three Araucariaceae genera possess *rrn5*-IRs, though their recombinant activity has not been assessed^51^. The diverse set of IRs ubiquitously associates with the presence of isomeric plastomes, which suggests convergent evolution of isomeric plastomes among cupressophyte families.

Our PCR and read mapping analyses show that the isomeric arrangements are not present in equal percentages. Instead, the major arrangements strikingly exceed the minor ones in their relative ratios (Figs 2; S2). This feature suggests that *trnQ*-IRs mediate recombination at low frequency in both *Pseudotaxus* and *Taxus*. In Taxaceae, *trnQ*-IR lengths are between 216 and 564 bp (Table 1). These lengths of repeats occasionally mediate recombination in mitochondria^52^. However, our data reveal that major isomeric arrangements have shifted among conspecifics in *T. brevifolia*, *T. floridana*, and *T. globosa*. Intraspecific shifts in major isomeric arrangements might also occur in *T. chinensis* because an earlier reported plastomes (arrangement A)^53,54^ and our newly assemblies (arrangement B: Figs 1 and 2) are oriented differently. Guo *et al*.^6^ proposed that major isomeric arrangements have shifted multiple times during the diversification of cupressophytes. This proposition is further extended by our findings that major and minor plastomic arrangements could shift at intraspecific levels. In mitochondria, selective amplification was thought to account for alternation of major isomers^55^. Nevertheless, it remains unclear whether accumulated mutations that benefit amplification would enable a minor isomer to become a major one in plastids. Unfortunately, the Illumina reads used in this study are too short to extensively quantitate mutations between isomers. The PacBio long-read sequencing technology that was recently used to distinguish heteroplastomic DNAs within individuals^56^ opens a new avenue to deepen our understanding of isomeric plastome evolution in future.

To date, using the entire plastome sequence as a super-barcode has been demonstrated to be useful for discriminating species in diverse lineages, such as rice^22^, cacao^25^, *Araucaria*^26^, and *Stipa*^57^, especially in some taxonomically complex groups^29,30^. Our ML tree inferred from the entire plastome sequence shows that all conspecific samples were resolved as monophyletic with robust support (Fig. 3), therefore the super-barcoding approach is validated for discriminating *Taxus* species. It has been proposed that the super-barcoding approach circumvents the issues of gene deletion, locus choice, and low PCR recovery rate often encountered in studies using conventional barcodes^26,45,58^. Despite sharing the same set of plastid genes (Table 1), the sampled *Taxus* species differ in their plastome organizations (i.e., arrangement A or B), which hampers the performance of whole plastome alignments. Identification of LCBs before conducting alignments is a prerequisite for using the super-barcoding approach in cupressophyte lineages whose plastomes are highly rearranged^51,59^. Collectively, the plastome as super-barcode showed a great promise for distinguishing closely related species in *Taxus*.

Lineage-specific barcodes are also thought to enhance the resolution of species discrimination because they might provide more sufficient information within a particular group than traditional barcodes^17^. Indeed, the core barcode *matK* + *rbcL* suggested by the CBOL Plant Working Group (2009) only distinguished 63% of our sampled *Taxus* species (Table S2). Although *trnL-trnF* showed high discriminatory rates for yew species, it did not discern two New World yews (i.e., *T. globosa* and *T. floridana*) based on the Tree-based method^41^. The combination of *trnL-trnF* and nrITS could effectively discriminate between all the yew species^41^. In this study, *accD*, *ycf1*, *ycf2*, and *rrn16-rrn23* are shown to successfully discriminate all species, with the former two containing distinct barcoding gaps. *rpoB* contains a barcoding gap, but did not yield a 100% species resolution. In contrast, all conspecific accessions formed monophyletic clades with a 100% support from both non-barcoding loci: *ycf2* and *rrn16-rrn23*. Collectively, our results imply that the existence of a clear barcoding gap is not prerequisite to discriminate species 100% successfully. Considering the length of the loci, we therefore suggest *accD* and *rrn16-rrn23* can be effective special barcoding loci for discriminating *Taxus* species. Exploration of potential barcodes/mutational hotspots is highly dependent on the estimated sequence divergence^60–63^. In *Taxus*, the locus *clpP* (Fig. 4) exhibits the highest degree of both intra- and inter-specific sequence divergences, indicating its potential for population genetics studies. However, *clpP* only achieved a discriminatory rate of 81.25% in our practical analysis (Table S2). As a result, we suggest that future researchers perform practical analyses in order to accurately evaluate the discriminatory power of selected loci in barcoding studies.

## Conclusion

Continuous advances in sequencing technologies make obtaining complete plastomes from major lineages across a genus more feasible. A total of 49 plastomes from *Pseudotaxus chienii* and 16 *Taxus* species were elucidated and compared in the present study. Our PCR and read mapping results together support the existence of *trnQ*-IR mediated isomeric plastomes in both *Pseudotaxus* and *Taxus*. We provide evidence, for the first time, that major isomeric arrangements have shifted among populations. We successfully used entire plastome sequences to distinguish all *Taxus* species, including three potential cryptic types, supporting that the plastome sequences themselves in *Taxus* species are effective super-barcodes for species identification and discovery. Moreover, four single loci—*accD*, *ycf1*, *ycf2*, and *rrn16-rrn23*—are capable of achieving 100% discriminatory rates; of these, *accD* and *rrn16-rrn23*, which have never been used before in discrimination of yews, are modest in length, and we therefore suggest that they can be used as special DNA barcodes for yews. Further studies should design primers and examine the PCR recovery rate for these four loci with a more comprehensive set of samples as our previous study^40^. In conclusion, our newly developed genetic resources of *Taxus* plastomes and barcoding candidates may aid in conservation and authentication of endangered *Taxus* species.

## Methods

### Plant materials, DNA extraction and sequencing

As *Taxus* is a taxonomically difficult genus and its interspecific classification remains controversial. This study adopts Farjon’s classification^31^ and follows our recent study^40^. A total of 16 *Taxus* species worldwide were sampled and identified on the basis of the morphological and molecular evidence described in our previous studies^38–41,64,65^, including 13 species (*T. brevifolia* Nutt., *T. globosa* Schltdl., *T. floridana* Nutt., *T. canadensis* Marshall, *T. cuspidata* Siebold & Zucc., *T. baccata* L., *T. contorta* Griff., *T. chinensis* (Pilg.) Rehd., *T. mairei* (Lemée & Lév.) S.Y. Hu, *T. wallichiana* Zucc., *T. calcicola* L.M. Gao & Mich. Möller, *T. florinii* Spjut, *T. phytonii* Spjut) and three potential cryptic species, of which two (Emei and Qingling types) have been previously described^39,41,64^ and one (i.e., Huangshan type) is newly discovered (it was formally treated as *T. chinensis*, described from high elevation mountains in eastern China).

To assess genetic variation at the intraspecific level, two to three individuals per species were sampled from different populations for each *Taxus species except T. canadensis and Qinling type*. In addition, two individuals of *Pseudotaxus chienii* were also sampled and used as the outgroup. The specimens and vouchers (Table S1) of these sampled taxa are deposited in the herbarium of Kunming Institute of Botany, Chinese Academy of Science (KUN), Yunnan, China.

Total genomic DNA was extracted from fresh or silica-gel dried leaves using a modified CTAB method^66^, in which 4% CTAB was used with incorporation of 0.1% DL-dithiothreitol (DTT). After it was quantified using Qubit 2.0 (Invitrogen, Carlsbad, CA, USA), the extracted DNA was sheared into approximately 500 bp fragments for library construction using standard protocols (NEBNext® Ultra II™DNA Library Prep Kit for Illumina®). All samples were sequenced on an Illumina HiSeq X Ten platform in CloudHealth Company (Shanghai, China) to generate approximately 5‒70 million paired-end 150 bp reads (Table S1).

### Plastome assembly and annotation

We used the GetOrganelle pipeline^67^ to *de novo* assemble plastomes. In this pipeline, plastomic reads were extracted from total genomic reads and were subsequently assembled using SPAdes version 3.10^68^. Plastid genes were annotated using Geneious 11.0.3^69^ with the published plastome of *T. mairei*^70^ as the reference. Transfer RNAs (tRNAs) were confirmed by their specific structure predicted by tRNAscan-SE 2.0^71^. Plastomes were visualized using Circos 0.67^72^.

### Locally co-linear block (LCB) identification and sequence alignment

The locally co-linear blocks (LCBs) between *Pseudotaxus* and *Taxus* plastomes were identified using progressMavus^73^ with the default options and *psbA* as the initial point. Sequences of LCBs or loci were aligned using MAFFT 7.0^74^. The parameter set was algorithm = auto, scoring matrix = 200PAM/k = 2, gap open penalty = 1.53, and offset value = 0.123.

### Tree construction and pairwise distance calculation

The alignments of LCBs were concatenated in DAMBE 5.0^75^. We used jModelTest2^76^ to assess the best models for tree construction under the corrected Akaike Information Criterion (AIC_c_). Maximum likelihood trees inferred from this concatenated alignment were analyzed under the GTRGAMMAI model with 1,000 rapid bootstrap searches in RAxML 8.2^77^. For each single locus or combined multiple loci, estimates of pairwise distances and neighbor-joining (NJ) trees were carried out in MEGA 7^78^ under the Kimura 2-parameter method. The bootstrap supports for NJ trees were computed with 1,000 replicates. All yielded tree topologies were condensed under a 50% majority rule in MEGA 7.

### Verification of isomeric arrangements

To verify isomeric arrangements, we adopted two approaches. First, semi-quantitative PCRs involved use of specific primers to yield amplicons unique to the isomeric arrangements (see Fig. 2A). PCR reactions were performed on a GeneAmp PCR System 9700 thermal cycler (PerkinElmer, Foster City, CA, USA). The 20 µL PCR mixture contained 1 µL total DNA (20 ng/µL), 0.5 µL each of the forward and reverse primers (10 µM), 10 µL Tiangen 2 × Taq PCR MasterMix (Tiangen Biotech, Beijing), and 8 µL ddH_2_O. After an initial denaturation at 94 °C for 5 min, PCR reactions were conducted for 20, 25, 30 and 35 cycles, respectively. Each cycle included 94 °C for 30 s, annealing at 56 °C for 30 s, and elongation at 72 °C for 1 min. The PCR procedure ended up with a final incubation at 72 °C for 7 min. PCR gel images were taken using a G:BOX gel doc system (SYNGENE, USA) under the exposure time of 1s500ms/1s800ms, followed by “color invert” in PhotoImpact 10 (https://www.paintshoppro.com/). Second, we mapped the Illumina paired-end reads onto the regions specific to each of the isomeric arrangements using Geneious with the default setting. Paired-end reads that spanned the entire *trnQ*-IR were counted if the sequence identity was >90%. The mapping scenario was viewed and checked in Geneious.

## Supplementary information

Supplementary file

## Data Availability

All DNA sequences have been deposited in GenBank with accession numbers MH390441 to MH390489 (Table S1).
