# Supplementary material for: Prevalence of isomeric plastomes and effectiveness of plastome super-barcodes in yews (Taxus) worldwide
Source: Sci Rep. 2019 Feb 26;9:2773. doi: 10.1038/s41598-019-39161-x (PMC6391452; doi:10.1038/s41598-019-39161-x)
Supplement: Supplementary file 1 — Supplementary file [file 41598_2019_39161_MOESM1_ESM.pdf]

Prevalence of isomeric plastomes and effectiveness of plastome super-barcodes in yews (*Taxus)* worldwide

Chao-Nan Fu[1,2,3]*, Chung-Shien Wu[4]*, Lin-Jiang Ye[2,3], Zhi-Qiong Mo[1,3], Jie Liu[1], Yu-Wen Chang[4], De-Zhu Li[2,3], Shu-Miaw Chaw[4]†, Lian-Ming Gao[1]†

[1]Key Laboratory for Plant Diversity and Biogeography in East Asia, Kunming Institute of Botany, Chinese Academy of Sciences, Kunming, Yunnan 650201, China

[2]Germplasm Bank of Wild Species, Kunming Institute of Botany, Chinese Academy of Sciences, Kunming, Yunnan 650201, China

[3]Kunming College of Life Science, University of Chinese Academy of Sciences, Kunming, Yunnan 650201, China

[4]Biodiversity Research Center, Academia Sinica, Taipei 11529, Taiwan

* These authors contributed equally to this paper.

† Corresponding authors: smchaw@sinica.edu.tw (SM Chaw), gaolm@mail.kib.ac.cn (LM Gao)

Running title: Comparative plastomics of yews

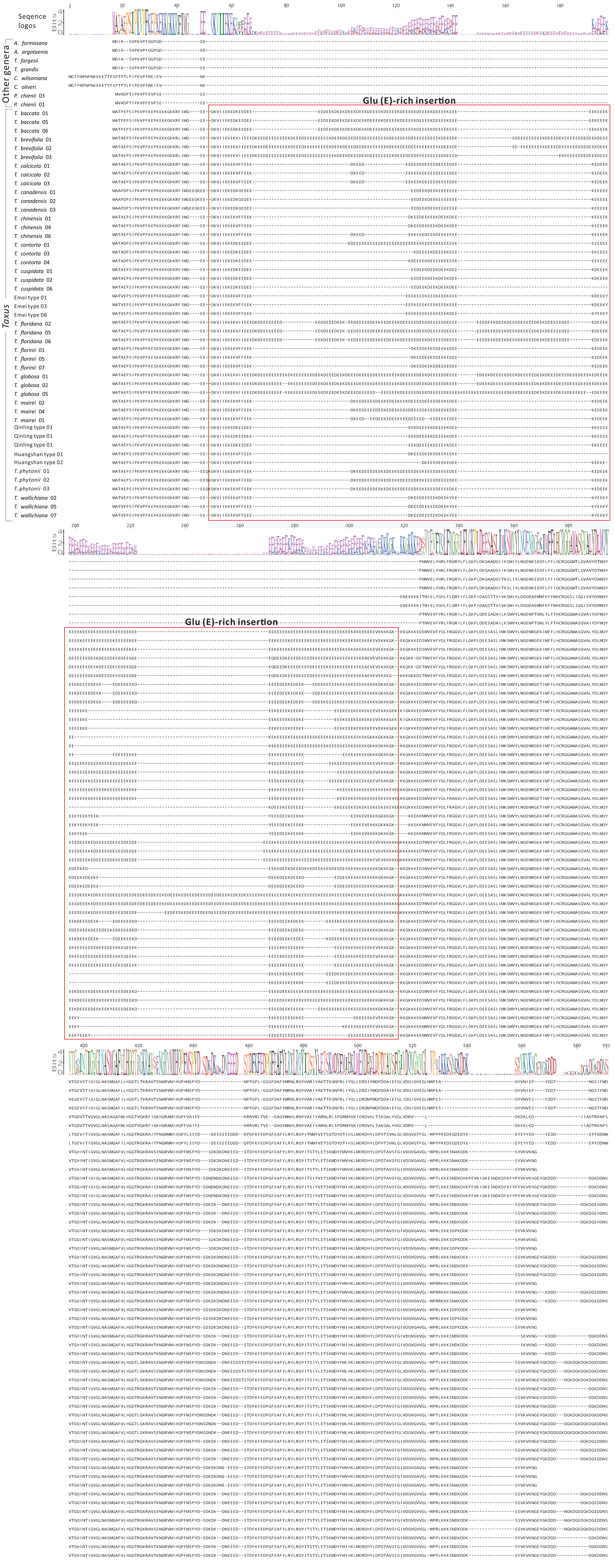

Figure S1. Amino acid alignment of clpP revealing a Taxus-specific Glu (E)-rich insertion. Sequence logos stand for the degree of sequence conservation at aligned positions.

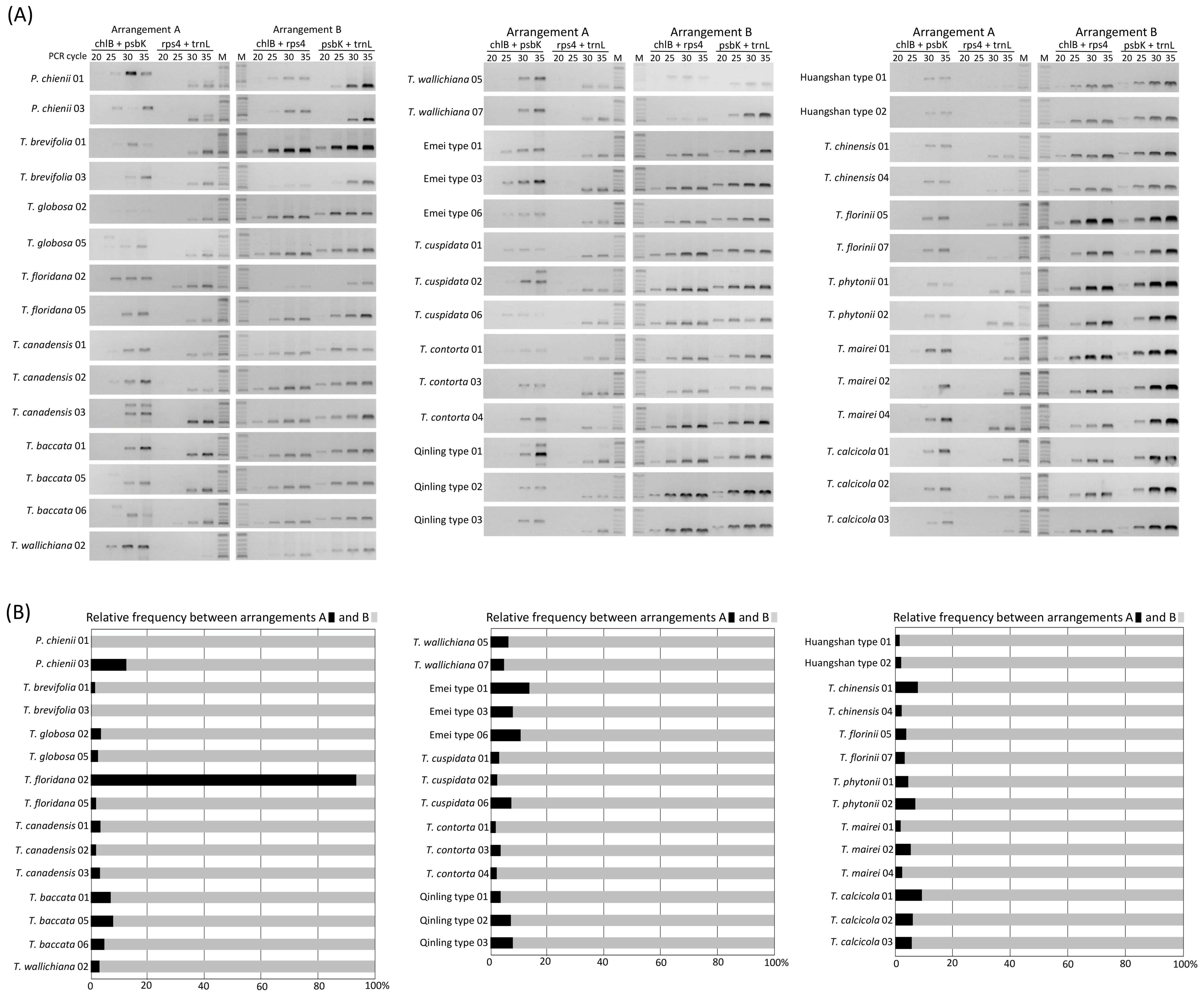

Figure S2. Examination of isomeric plastome arrangements A and B in *Pseudotaxus* and *Taxus* based on PCR (A) and read mapping (B) analyses.

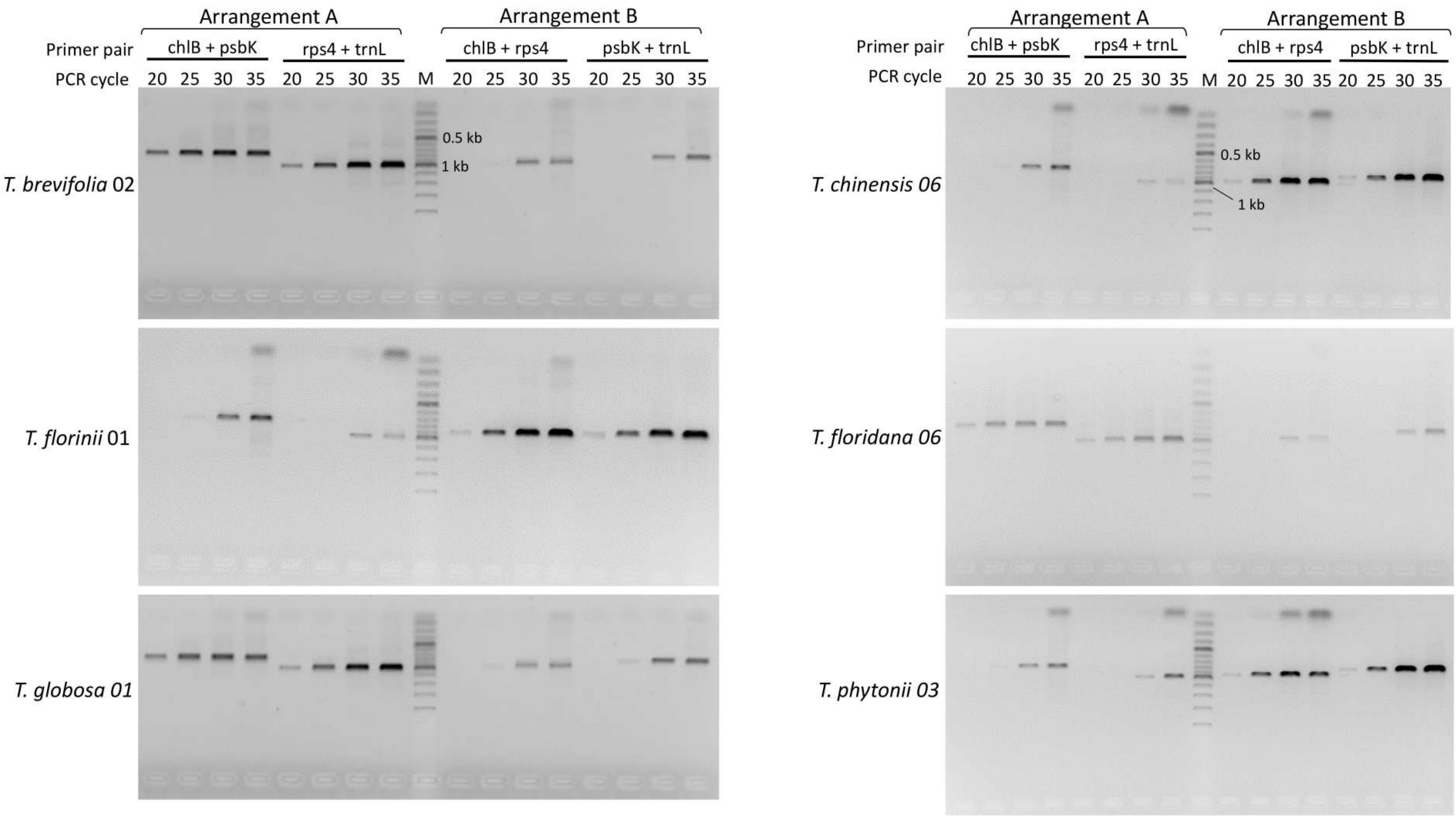

Figure S3. Full-length gels showing the existence of the isomeric arrangements A and B in *T. brevifolia* 01, *T. florinii* 01, *T. globose* 01, *T. chinensis* 06, *T. floridana* 06, and *T. phytonii* 03.

Table S1 Voucher numbers, GenBank accessions, and NGS performance for the 49 sequenced samples in this study

Genus	Sampled individual	Voucher number	GenBank accession No.	No. of reads aftering trimming	No. of mapped reads	Sequencing coverage (mean)
Pseudotaxus	*Pseudotaxus chienii* 01	GLM133982	MH390460	9,529,376	64,583	74
	Pseudotaxus chienii 03	JXAU-PC3	MH390485	10,405,954	129,434	148
Taxus	*Taxus baccata* 01	Wuzy-2013001	MH390464	16,400,846	340,548	397
	Taxus baccata 05	T1	MH390453	8,557,656	147,975	207
	Taxus baccata 06	T2	MH390454	8,462,156	187,211	218
	Taxus brevifolia 01	938|2013*B	MH390457	20,575,860	634,596	736
	Taxus brevifolia 02	938|2013*C	MH390484	60,570,338	2,269,162	2,716
	Taxus brevifolia 03	3870	MH390459	5,354,624	35,457	41
	Taxus calcicola 01	GLM164262	MH390451	32,662,650	608,516	719
	Taxus calcicola 02	GLM123951	MH390461	24,297,058	298,327	347
	Taxus calcicola 03	GLM123950	MH390489	12,320,206	429,845	501
	Taxus canadensis 01	Marc Cadotte 1	MH390483	38,289,258	1,012,623	1,185
	Taxus canadensis 02	Marc Cadotte 2	MH390448	23,933,626	510,897	596
	Taxus canadensis 03	Marc Cadotte 3	MH390466	23,596,588	601,184	698
	Taxus chinensis 01	TCP0102	MH390476	6,695,992	148,994	173
	Taxus chinensis 04	TCP0202	MH390478	5,264,518	125,623	172
	Taxus chinensis 06	TCP0303	MH390442	5,110,812	52,120	61
	Taxus contorta 01	GLM123764	MH390455	8,059,742	161,893	211
	Taxus contorta 03	Pakistan-47	MH390443	10,943,434	106,576	132
	Taxus contorta 04	Pakistan-51	MH390449	34,299,304	714,206	837
	Taxus cuspidata 01	GLM164311	MH390465	8,392,846	247,503	289
	Taxus cuspidata 02	GLM164312	MH390447	15,612,234	464,678	551
	Taxus cuspidata 06	GLM164316	MH390477	10,425,646	298,011	348
	Taxus floridana 02	Kevin 2	MH390480	64,824,584	880,860	1,018
	Taxus floridana 05	AB620111104	MH390474	12,339,464	129,018	149
	Taxus floridana 06	2011111109	MH390468	7,955,656	333,559	384
	Taxus florinii 01	TFP0302	MH390487	18,508,852	155,754	189
	Taxus florinii 05	TFP0402	MH390463	25,574,090	832,299	1,118
	Taxus florinii 07	ML04	MH390473	15,181,156	98,793	125
	Taxus globosa 01	1M19	MH390488	17,817,588	349,863	408
	Taxus globosa 02	4M1	MH390467	6,003,810	196,387	241
	Taxus globosa 05	AB6	MH390450	39,286,476	989,602	1,147
	Taxus mairei 01	LJ-06091	MH390458	7,388,776	76,220	89
	Taxus mairei 02	JXAU-Taxus2	MH390479	47,437,048	942,353	1,094
	Taxus mairei 04	JXAU-Taxus4	MH390482	6,533,868	74,955	87
	Taxus phytonii 01	829	MH390470	6,901,366	146,967	171
	Taxus phytonii 02	1254	MH390445	7,408,502	41,966	49
	Taxus phytonii 03	BH6	MH390441	11,326,008	100,783	118
	Taxus wallichiana 02	TWP0102	MH390452	6,632,504	62,967	74
	Taxus wallichiana 05	TWP0202	MH390462	16,096,328	624,127	947
	Taxus wallichiana 07	CY10	MH390446	13,898,538	180,543	211
	**Emei type 01**	TEP0101	MH390456	5,869,710	36,963	43
	**Emei type 03**	TEP0202	MH390472	74,524,874	655,584	854
	**Emei type 06**	TEP0303	MH390475	13,030,724	205,738	326
	**Huangshan type 01**	GLM133979	MH390469	13,896,690	303,194	354
	**Huangshan type 02**	GLM133986	MH390486	39,466,900	1,438,968	1,681
	**Qinling type 01**	QL1	MH390444	15,694,094	298,466	459
	**Qinling type 02**	QL2	MH390481	6,875,442	254,119	326
	**Qinling type 03**	QL3	MH390471	12,706,482	292,202	382

Sampled individuals in blod are cryptic species

Table S2 Examination of 44 protein-coding genes and 29 intergenic spacers for discriminating the 16 species of Taxus

Locus / Gene[1]	Length (bp)[2]	Interspecific distance (%) Min	Interspecific distance (%) Max	Intraspecific distance (%) Min	Intraspecific distance (%) Max	Overlap[3]	Taxon[4] *T. brevifolia*	*T. globosa*	*T. floridana*	Emei type	*T. wallichiana*	*T. canadensis*	*T. cuspidata*	*T. baccata*	*T. contorta*	Qinling type	Huangshan type	*T. chinensis*	*T. florinii*	*T. phytonii*	*T. calcicola*	*T. mairei*	Discriminatory power (%)
accD	2214.6 ± 41	0.09%	6.79%	0.00%	0.05%	No	100	66	98	100	96	100	100	99	100	100	97	99	99	100	56	85	100.00%
atpA	1521 ± 0	0.00%	0.46%	0.00%	0.00%	Yes	95	n.d.	n.d.	n.d.	n.d.	n.d.	86	n.d.	64	n.d.	n.d.	n.d.	n.d.	89	n.d.	n.d.	25.00%
atpB	1473 ± 0	0.00%	0.55%	0.00%	0.14%	Yes	98	n.d.	n.d.	61	n.d.	n.d.	65	90	63	n.d.	64	n.d.	65	63	n.d.	n.d.	50.00%
*atpF**	1245 ± 3.7	0.00%	0.89%	0.00%	0.00%	Yes	95	n.d.	68	65	62	62	n.d.	63	n.d.	69	64	62	95	64	n.d.	65	75.00%
atpI	744 ± 0	0.00%	0.54%	0.00%	0.13%	Yes	64	n.d.	n.d.	n.d.	n.d.	n.d.	n.d.	n.d.	n.d.	65	64	65	87	n.d.	n.d.	n.d.	31.25%
ccsA	953.2 ± 2	0.00%	0.95%	0.00%	0.00%	Yes	99	n.d.	n.d.	63	n.d.	61	n.d.	n.d.	64	n.d.	62	64	86	n.d.	n.d.	n.d.	43.75%
cemA	783 ± 0	0.00%	0.77%	0.00%	0.00%	Yes	86	n.d.	64	66	58	87	n.d.	89	n.d.	n.d.	88	n.d.	64	n.d.	n.d.	n.d.	50.00%
chlB	1548 ± 0	0.00%	0.65%	0.00%	0.06%	Yes	84	n.d.	n.d.	n.d.	n.d.	86	n.d.	84	n.d.	n.d.	n.d.	62	66	65	n.d.	64	43.75%
chlL	870.4 ± 1	0.00%	1.39%	0.00%	0.00%	Yes	n.d.	n.d.	n.d.	96	82	n.d.	n.d.	n.d.	n.d.	n.d.	62	79	66	65	90	77	50.00%
chlN	1397 ± 10	0.00%	1.60%	0.00%	0.07%	Yes	100	69	n.d.	100	99	60	89	68	n.d.	65	63	53	n.d.	n.d.	61	n.d.	68.75%
clpP	1166.4 ± 171	0.29%	9.14%	0.00%	2.01%	Yes	100	n.d	100	100	99	100	98	100	79	93	n.d.	100	100	100	100	n.d.	81.25%
matK	1527.6 ± 5	0.00%	1.19%	0.00%	0.07%	Yes	100	n.d	n.d.	n.d.	n.d.	94	n.d.	71	52	66	n.d.	64	97	60	n.d.	n.d.	50.00%
*ndhA**	1856.5 ± 8.5	0.00%	0.54%	0.00%	0.00%	Yes	90	n.d.	65	n.d.	n.d.	95	62	63	62	64	95	n.d.	90	n.d.	n.d.	n.d.	56.25%
*ndhB**	2198.4 ± 7.4	0.05%	1.67%	0.00%	0.09%	Yes	100	100	68	86	96	95	98	96	n.d.	99	89	97	n.d.	92	79	95	87.50%
ndhD	1503 ± 0	0.00%	0.47%	0.00%	0.00%	Yes	94	n.d.	n.d.	n.d.	n.d.	n.d.	n.d.	n.d.	63	n.d.	n.d.	66	n.d.	n.d.	n.d.	n.d.	18.75%
ndhF	2229.1 ± 4	0.00%	1.31%	0.00%	0.04%	Yes	100	n.d.	n.d.	54	54	95	69	n.d.	64	n.d.	63	92	98	86	83	63	75.00%
ndhG	540 ± 0	0.00%	0.56%	0.00%	0.00%	Yes	n.d.	n.d.	n.d.	n.d.	n.d.	n.d.	n.d.	n.d.	63	n.d.	n.d.	65	n.d.	n.d.	n.d.	n.d.	12.50%
ndhH	1179 ± 0	0.00%	0.51%	0.00%	0.08%	Yes	89	n.d.	n.d.	n.d.	n.d.	63	n.d.	n.d.	n.d.	n.d.	n.d.	n.d.	n.d.	62	n.d.	63	25.00%
ndhI	488.8 ± 1	0.00%	0.82%	0.00%	0.00%	Yes	66	n.d.	n.d.	66	n.d.	n.d.	n.d.	n.d.	n.d.	n.d.	n.d.	n.d.	n.d.	n.d.	n.d.	n.d.	12.50%
ndhJ	477.3 ± 12	0.00%	0.42%	0.00%	0.00%	Yes	64	n.d.	n.d.	64	n.d.	n.d.	n.d.	63	n.d.	n.d.	n.d.	n.d.	n.d.	n.d.	n.d.	n.d.	18.75%
ndhK	762.4 ± 2	0.00%	0.66%	0.00%	0.13%	Yes	87	n.d.	n.d.	n.d.	66	64	n.d.	n.d.	n.d.	n.d.	n.d.	62	59	78	n.d.	n.d.	37.50%
petA	963 ± 0	0.00%	0.52%	0.00%	0.10%	Yes	79	n.d.	n.d.	63	n.d.	n.d.	65	n.d.	n.d.	87	n.d.	n.d.	n.d.	n.d.	n.d.	n.d.	25.00%
*petB**	1427.4 ± 5.1	0.00%	0.99%	0.00%	0.14%	Yes	97	n.d.	n.d.	82	93	n.d.	86	n.d.	95	99	n.d.	77	96	n.d.	n.d.	n.d.	50.00%
*petD**	1154.7 ± 1.8	0.00%	1.05%	0.00%	0.09%	Yes	99	n.d.	n.d.	78	n.d.	96	85	n.d.	61	63	86	62	n.d.	n.d.	n.d.	65	56.25%
psaA	2250 ± 0	0.00%	0.36%	0.00%	0.04%	Yes	62	n.d.	n.d.	n.d.	63	n.d.	63	n.d.	64	n.d.	n.d.	62	89	n.d.	n.d.	n.d.	37.50%
psaB	2202 ± 0	0.00%	0.32%	0.00%	0.05%	Yes	98	n.d.	n.d.	65	n.d.	63	n.d.	88	n.d.	n.d.	n.d.	n.d.	88	n.d.	n.d.	63	37.50%
psbA	1059 ± 0	0.00%	0.38%	0.00%	0.09%	Yes	81	n.d.	n.d.	n.d.	n.d.	64	n.d.	n.d.	n.d.	n.d.	n.d.	n.d.	n.d.	n.d.	n.d.	n.d.	12.50%
psbB	1524 ± 0	0.00%	0.73%	0.00%	0.00%	Yes	95	n.d.	66	61	n.d.	93	n.d.	96	n.d.	n.d.	n.d.	n.d.	n.d.	n.d.	n.d.	n.d.	31.25%
psbC	1419 ± 0	0.00%	0.57%	0.00%	0.07%	Yes	86	n.d.	n.d.	n.d.	n.d.	n.d.	n.d.	n.d.	65	n.d.	62	64	n.d.	n.d.	n.d.	n.d.	25.00%
psbD	1059 ± 0	0.00%	0.19%	0.00%	0.00%	Yes	n.d.	n.d.	n.d.	n.d.	n.d.	n.d.	n.d.	n.d.	n.d.	n.d.	n.d.	n.d.	n.d.	61	n.d.	n.d.	6.25%
rbcL	1425 ± 0	0.00%	0.40%	0.00%	0.00%	Yes	91	n.d.	n.d.	n.d.	n.d.	65	73	63	64	88	66	53	84	64	n.d.	n.d.	62.50%
*rpl16**	1291 ± 8	0.00%	1.25%	0.00%	0.00%	Yes	99	65	86	99	61	99	n.d.	n.d.	64	61	64	n.d.	95	63	n.d.	n.d.	68.75%
*rpl2**	1456.6 ± 12	0.00%	1.47%	0.00%	0.07%	Yes	100	n.d.	n.d.	81	59	100	95	67	63	n.d.	81	n.d.	63	n.d.	n.d.	n.d.	56.25%
rpoA	987.6 ± 13	0.00%	1.02%	0.00%	0.00%	Yes	87	n.d.	n.d.	92	n.d.	75	n.d.	64	n.d.	66	94	n.d.	85	n.d.	64	n.d.	50.00%
rpoB	3275.8 ± 0.7	0.03%	0.67%	0.00%	0.00%	No	99	nd	68	100	96	98	94	86	63	93	64	65	n.d.	66	89	61	87.50%
*rpoC1**	2802.3 ± 8.6	0.04%	0.90%	0.00%	0.04%	Yes	100	66	n.d.	96	99	81	97	n.d.	n.d.	78	88	85	88	98	94	n.d.	75.00%
rpoC2	3021.1 ± 4.5	0.00%	0.93%	0.00%	0.03%	Yes	100	n.d.	n.d	68	n.d.	99	85	76	94	88	99	86	93	87	88	n.d.	75.00%
rps11	443 ± 0	0.00%	1.82%	0.00%	0.00%	Yes	97	n.d.	n.d.	n.d.	n.d.	62	n.d.	n.d.	n.d.	87	n.d.	66	n.d.	68	n.d.	n.d.	31.25%
rps2	699 ± 0	0.00%	1.01%	0.00%	0.14%	Yes	86	n.d.	61	89	n.d.	62	n.d.	n.d.	89	n.d.	n.d.	62	86	86	n.d.	n.d.	50.00%
rps3	704.6 ± 1.5	0.00%	1.87%	0.00%	0.28%	Yes	100	67	n.d.	65	n.d.	84	66	63	97	n.d.	n.d.	n.d.	n.d.	84	n.d.	n.d.	50.00%
rps4	621 ± 0	0.00%	0.65%	0.00%	0.00%	Yes	81	n.d.	n.d.	n.d.	n.d.	64	n.d.	n.d.	n.d.	64	n.d.	65	67	n.d.	n.d.	n.d.	31.25%
rps7	468 ± 0	0.00%	1.73%	0.00%	0.00%	Yes	98	66	n.d.	n.d.	64	n.d.	n.d.	n.d.	n.d.	n.d.	n.d.	n.d.	n.d.	62	n.d.	n.d.	25.00%
ycf1	6740 ± 50.8	0.10%	3.55%	0.00%	0.08%	No	100	64	98	100	100	100	100	100	100	100	100	100	100	100	100	99	100.00%
ycf2	7348.9 ± 415.1	0.18%	5.56%	0.00%	0.91%	Yes	100	100	100	100	100	100	100	100	100	100	100	100	100	100	100	100	100.00%
Intergenic spacer																							
atpE-rbcL	502 ± 18.8	0.00%	2.88%	0.00	0.00	Yes	100	n.d.	n.d.	100	89	n.d.	n.d.	67	n.d.	n.d.	n.d.	97	95	81	n.d.	64	50.00%
atpI-atpH	617.4 ± 10.7	0.00%	2.01%	0.00	0.00	Yes	95	n.d.	99	86	n.d.	88	87	n.d.	63	79	n.d.	87	n.d.	64	n.d.	n.d.	62.50%
clpP-infA	453.8 ± 35	0.00%	2.54%	0.00	0.00	Yes	98	n.d.	n.d.	n.d.	87	98	n.d.	91	43	91	n.d.	n.d.	n.d.	64	61	n.d.	50.00%
ndhC-trnV	806.7 ± 6.7	0.00%	2.19%	0.00	0.00	Yes	100	n.d.	63	96	66	100	63	88	87	88	99	99	95	70	n.d.	n.d.	81.25%
ndhF-rpl32	808.5 ± 20.7	0.00%	1.26%	0.00	0.00	Yes	97	n.d.	n.d.	87	n.d.	n.d.	69	96	77	95	n.d.	84	63	81	53	n.d.	62.50%
petA-psbJ	446.9 ± 24.5	0.00%	3.27%	0.00	0.00	Yes	89	n.d.	n.d.	83	78	87	65	61	64	87	n.d.	n.d.	93	64	n.d.	n.d.	62.50%
petN-psbM	738.4 ± 38.6	0.00%	2.96%	0.00	0.01	Yes	100	n.d.	n.d.	99	n.d.	97	n.d.	n.d.	n.d.	97	89	62	51	81	n.d.	n.d.	50.00%
psaI-ycf4	569.6 ± 11.5	0.00%	2.58%	0.00	0.00	Yes	100	n.d.	n.d.	n.d.	n.d.	86	62	n.d.	63	50	n.d.	n.d.	n.d.	n.d.	n.d.	n.d.	31.25%
psbD-trnT	1225.6 ± 71.7	0.00%	1.46%	0.00	0.00	Yes	100	64	n.d.	96	84	n.d.	65	64	n.d.	64	n.d.	73	73	62	n.d.	65	68.75%
psbE-petL	1209.3 ± 213	0.00%	2.51%	0.00	0.00	Yes	88	n.d.	79	n.d.	70	99	95	73	92	90	83	99	99	63	97	94	87.50%
psbI-psbK	646 ± 8.9	0.00%	2.53%	0.00	0.00	Yes	100	n.d.	n.d.	91	n.d.	100	70	97	97	99	76	99	98	98	n.d.	86	75.00%
psbM-trnC	500.7 ± 5.9	0.00%	1.63%	0.00	0.00	Yes	95	n.d.	n.d.	64	n.d.	63	63	85	n.d.	87	n.d.	66	97	65	n.d.	n.d.	56.25%
rbcL-accD	472.2 ± 16.1	0.00%	2.59%	0.00	0.00	Yes	97	n.d.	n.d.	72	92	66	n.d.	86	90	n.d.	n.d.	n.d.	n.d.	n.d.	n.d.	80	43.75%
rpl20-5'rps12	730.1 ± 6.2	0.00%	1.54%	0.00	0.00	Yes	99	n.d.	64	n.d.	64	n.d.	98	n.d.	66	n.d.	98	65	98	n.d.	n.d.	64	56.25%
rpl32-trnP	960.9 ± 31.3	0.00%	1.84%	0.00	0.00	Yes	100	n.d.	65	84	n.d.	92	74	88	77	n.d.	87	63	93	87	66	n.d.	75.00%
rps15-ycf1	807.7 ± 82.5	0.00%	1.87%	0.00	0.00	Yes	100	n.d.	n.d.	n.d.	n.d.	99	69	95	n.d.	96	97	95	n.d.	n.d.	n.d.	n.d.	43.75%
rrn16-rrn23	1963.1 ± 160.8	0.27%	9.90%	0.00	0.02	Yes	100	51	92	99	100	100	100	100	62	98	100	100	88	98	100	66	100.00%
trnC-rpoB	738.2 ± 44.1	0.00%	4.28%	0.00	0.00	Yes	100	n.d.	n.d.	67	93	100	n.d.	75	74	n.d.	99	n.d.	95	n.d.	n.d.	n.d.	50.00%
trnD-petN	845.3 ± 12.6	0.00%	3.55%	0.00	0.01	Yes	100	n.d.	67	98	66	99	97	85	57	94	99	92	94	88	n.d.	n.d.	81.25%
trnF-ndhJ	514.5 ± 19.4	0.00%	3.03%	0.00	0.00	Yes	99	68	n.d.	89	n.d.	95	n.d.	64	63	87	65	n.d.	87	93	65	65	75.00%
trnI-ycf2	874.1 ± 51.6	0.11%	6.51%	0.00	0.00	Yes	100	100	53	93	100	99	92	97	100	100	97	97	88	73	n.d.	95	93.75%
trnK-chlB	1572.4 ± 29.3	0.07%	2.13%	0.00	0.00	Yes	100	85	86	87	94	100	99	65	53	96	n.d.	89	100	89	65	n.d.	87.50%
trnL-ndhB	515.9 ± 20.3	0.00%	6.43%	0.00	0.00	Yes	100	n.d.	n.d.	96	54	99	n.d.	95	100	52	99	n.d.	80	66	69	88	75.00%
trnN-ndhF	531.5 ± 30.1	0.00%	2.61%	0.00	0.00	Yes	100	60	n.d.	91	80	64	n.d.	64	n.d.	n.d.	n.d.	97	n.d.	80	n.d.	n.d.	50.00%
trnR-psaM	512.4 ± 80.7	0.00%	2.99%	0.00	0.00	Yes	72	n.d.	n.d.	99	n.d.	94	86	65	n.d.	99	91	96	n.d.	n.d.	n.d.	n.d.	50.00%
trnR-trnN	457.6 ± 10.9	0.00%	6.22%	0.00	0.00	Yes	100	51	n.d.	94	76	97	98	n.d.	91	76	68	n.d.	97	58	n.d.	n.d.	68.75%
trnT-trnE	862.1 ± 42.6	0.00%	3.09%	0.00	0.01	Yes	98	79	n.d.	96	84	n.d.	95	63	54	n.d.	n.d.	61	95	65	n.d.	65	68.75%
ycf1-chlN	914.3 ± 128.5	0.00%	8.15%	0.00	0.03	Yes	100	n.d.	56	71	99	99	100	69	92	100	98	100	96	n.d.	82	n.d.	81.25%
ycf3-psaA	550.1 ± 9.1	0.00%	2.58%	0.00	0.00	Yes	99	n.d.	n.d.	n.d.	n.d.	59	96	n.d.	n.d.	n.d.	n.d.	63	n.d.	99	64	n.d.	37.50%

[1]Intron-containing genes are labled with a "*".

[2]Mean ± standard deviation.

[3]Intra- and inter-specifc K2P distances are overlapped in distribution.

[4]Numbers indiacte bootstrap values (%) that supported a monophyletic clade of conspecific accessions; "n.d." denotes taxa not distinguished or bootstrap values smaller than 50%, despite formation of a monophyletic clade.
